# Familial coaggregation and shared familiality of functional and internalizing disorders in the Lifelines cohort

**DOI:** 10.1017/S003329172500100X

**Published:** 2025-05-02

**Authors:** Martje Bos, Rei Monden, Naomi R. Wray, Yiling Zhou, Kenneth S. Kendler, Judith G. M. Rosmalen, Hanna M. van Loo, Harold Snieder

**Affiliations:** 1 Department of Psychiatry, University of Groningen, University Medical Center Groningen, Groningen, The Netherlands; 2 Informatics and Data Science Program, Graduate School of Advanced Science and Engineering, Hiroshima University, Hiroshima, Japan; 3 Institute for Molecular Bioscience, The University of Queensland, Brisbane, QLD, Australia; 4 Department of Psychiatry and Big Data Institute, University of Oxford, Oxford, UK; 5 Department of Epidemiology, University of Groningen, University Medical Center Groningen, Groningen, The Netherlands; 6 Virginia Institute for Psychiatric and Behavioral Genetics, Virginia Commonwealth University, Richmond, VA, USA; 7 Department of Psychiatry, Virginia Commonwealth University, Richmond, VA, USA; 8 Department of Internal Medicine, University of Groningen, University Medical Center Groningen, Groningen, The Netherlands

**Keywords:** familial transmission, functional somatic syndromes, genetic correlations, heritability, mood and anxiety disorders

## Abstract

**Background:**

Functional disorders (FDs) are characterized by persistent somatic symptoms and are highly comorbid with internalizing disorders (IDs). To provide much-needed insight into FD etiology, we evaluated FD and ID familial coaggregation and shared familiality.

**Methods:**

Lifelines is a three-generation cohort study, which assessed three FDs (myalgic encephalomyelitis/chronic fatigue syndrome [ME/CFS], irritable bowel syndrome [IBS], and fibromyalgia [FM]) and six IDs (major depressive disorder [MDD], dysthymia [DYS], generalized anxiety disorder [GAD], agoraphobia [AGPH], social phobia [SPH], and panic disorder [PD]) according to diagnostic criteria. Based on 153,803 individuals, including 90,397 with a first-degree relative in Lifelines, we calculated recurrence risk ratios (λ_R_s) and tetrachoric correlations to evaluate familial aggregation and coaggregation of these disorders in first-degree relatives. We then estimated their familiality and familial correlations.

**Results:**

Familial aggregation was observed across disorders, with λ_R_ ranging from 1.45 to 2.23 within disorders and from 1.17 to 1.94 across disorders. Familiality estimates ranged from 22% (95% confidence interval [CI]: 16–29) for IBS to 42% (95% CI: 33–50) for ME/CFS. Familial correlations ranged from +0.37 (95% CI: 0.24–0.51) between FM and AGPH to +0.97 (95% CI: 0.80–1) between ME/CFS and FM. The highest familial correlation between an ID and FD was +0.83 (95% CI: 0.66–0.99) for MDD and ME/CFS.

**Conclusions:**

There is a clear familial component to FDs, which is partially shared with IDs. This suggests that IDs and FDs share both genetic and family-environmental risk factors. Of the FDs, ME/CFS is most closely related to IDs.

## Introduction

Functional disorders (FDs) are characterized by persistent somatic symptoms of unknown origin. In the absence of reproducibly observable pathophysiological processes, these disorders are diagnosed solely by symptoms. FDs are common (Haller, Cramer, Lauche, & Dobos, 2015; Nimnuan, Hotopf, & Wessely, 2001), costly (Konnopka et al., 2012) and disabling (Joustra, Janssens, Bültmann, & Rosmalen, 2015). The three best-known FDs are myalgic encephalomyelitis/chronic fatigue syndrome (ME/CFS), irritable bowel syndrome (IBS), and fibromyalgia (FM), with estimated prevalences of 1.5%, 9.1%, and 1.8%, respectively (Heidari, Afshari, & Moosazadeh, 2017; Lim et al., 2020; Rometsch et al., 2024). Understanding of FD etiology is still limited, but both biological and psychosocial factors have been associated with these disorders (Kleinstäuber et al., 2023).

There is evidence for a genetic component to FDs, with twin and genetic studies finding moderate levels of heritability for ME/CFS (Buchwald et al., 2001), IBS (Svedberg, Johansson, Wallander, & Pedersen, 2008), and FM (Dutta et al., 2020; Magnusson, Turkiewicz, Rydén, & Englund, 2024). Moreover, a family study observed heritable overlap between these FDs (Allen-Brady, Fyer, & Weissman, 2023). To increase understanding of FD etiology, further clarification of the genetic liability to FDs is important. However, genome-wide association studies (GWASs) of FDs are still in the early stages and have limited power (Bonfiglio et al., 2018; Hajdarevic et al., 2022; Moscati et al., 2023). Studying genetic liability shared with related disorders can provide further insight into the genetics of FDs.

Due to their high comorbidity with FDs, internalizing disorders (IDs) are good candidates to study alongside FDs. For instance, individuals who meet diagnostic criteria for ME/CFS, IBS, or FM show higher rates of major depressive disorder (MDD) (odds ratios [ORs] = 3.87–12.62) and generalized anxiety disorder (GAD) (ORs = 3.19–9.81) than those who do not meet FD diagnostic criteria (Thomas et al., 2024). Furthermore, a twin study suggests that FD-ID comorbidity is partly due to shared genetic factors (Kato, Sullivan, Evengård, & Pedersen, 2009). Similarly, a study using Swedish registry data found that individuals diagnosed with ME/CFS, IBS, or FM have an increased familial genetic risk for IDs (Kendler et al., 2023). Moreover, genetic loci associated with IBS have also been associated with depression and anxiety (Eijsbouts et al., 2021; Tavares et al., 2024; Tesfaye et al., 2023). Thus, studying both the genetics and the genetic relationships of IDs and FDs may advance the understanding of the etiology of both types of disorders.

Family studies provide a useful approach to study the vulnerability shared between disorders. Two such studies explored familial coaggregation of multiple FDs and IDs, both finding coaggregation between FM and MDD (Allen-Brady et al., 2023; Hudson et al., 2004). One study also observed coaggregation across ME/CFS, IBS, FM, and panic disorder (PD) (Allen-Brady et al., 2023). However, these studies either used medical records to define cases (Allen-Brady et al., 2023) or recruited patients from medical centers (Hudson et al., 2004). Both methods risk oversampling severe cases and may be influenced by help-seeking behavior and diagnostic biases (Tattan et al., 2024). The current study aims to assess familial aggregation, coaggregation, familiality, and familial correlations of IDs and FDs. Familial aggregation and coaggregation examine whether diseases cluster together in families. Familiality, a concept closely related to heritability, is the proportion of phenotypic variation attributable to familial effects, that is genetic and shared environmental effects combined. Familial correlation is a measure of how much familial effects on one trait overlap with familial effects on another trait. This study is performed in the large population-based Lifelines cohort (N = 153,803), which assessed FDs and IDs according to official diagnostic criteria.

## Methods

### Data

This study was conducted within the Lifelines cohort study. Lifelines is a multidisciplinary, prospective, population-based cohort study examining in a unique three-generation design the health and health-related behaviors of 167,729 persons living in the North of the Netherlands. It employs a broad range of investigative procedures to assess biomedical, sociodemographic, behavioral, physical, and psychological factors that contribute to health and disease in the general population, with a special focus on multimorbidity and complex genetics. Since 2006, three assessment waves have been completed (Sijtsma et al., 2022) in 2006–2013 (wave 1), 2014–2017 (wave 2), and 2019–2023 (wave 3). The current study used complete data from waves 1 and 2 as well as all data released from wave 3 up to 1 March 2024. The 153,803 adult participants with measurements on IDs or FDs during any of the three assessment waves were included in this study (Supplementary Figure 1).

The Lifelines cohort study followed the guidelines of the Declaration of Helsinki, and all procedures involving human subjects were approved by the Medical Ethical Committee of the University Medical Center Groningen. Furthermore, written informed consent was obtained from all participants.

### Measurements

#### Internalizing disorders

In all three waves, current MDD, dysthymia (DYS), GAD, agoraphobia (AGPH), social phobia (SPH), and PD were evaluated using the Mini-International Neuropsychiatric Interview (MINI) (Sheehan et al., 1998), which assesses these disorders per DSM-IV criteria (American Psychiatric Association, 1998). In wave 1, the MINI was administered as a face-to-face interview by a trained research nurse at a Lifelines research facility. In wave 2, participants completed a digital MINI questionnaire at the facility. In wave 3 used a digital MINI questionnaire emailed to participants for completion at home. The presence of MDD, DYS, and GAD required symptoms in the past two weeks, two years, and six months, respectively. The presence of AGPH, SPH, and PD required symptoms in the past month (van Loo et al., 2023), conforming to DSM-IV-TR duration criteria (American Psychiatric Association, 2000). Participants meeting the diagnostic criteria during any wave were labeled as cases; those who never met the criteria were labeled as controls. Thus, diagnostic status was determined based on assessments at up to three specific time points and does not include lifetime diagnoses.

#### Functional disorders

ME/CFS disease status was assessed according to the 1994 Centers for Disease Control and Prevention (CDC) diagnostic criteria (Fukuda et al., 1994). IBS status was assessed using ROME III criteria (Drossman, 2006), but the criteria related to symptom occurrence were adjusted to align with ROME IV criteria (Drossman, 2016). Thus, IBS was present if abdominal discomfort occurred more than once weekly, rather than three days a month, for over six months, in addition to two additional symptoms. FM was assessed according to the 2010 American College of Rheumatology (ACR) criteria (Wolfe et al., 2010). FDs were only assessed in Lifelines’ second and third waves. Participants meeting the diagnostic criteria during any wave were labeled as cases; those who did not were labeled as controls. Thus, diagnostic status was determined based on assessments at up to two specific time points and does not include lifetime diagnoses.

### Statistical analyses

#### Familial aggregation and coaggregation

Recurrence risk ratios (λ_R_s) were calculated to assess familial aggregation and coaggregation (Risch, 1990). λ_R_ is the ratio of disease prevalence in relatives of affected participants to the prevalence in the general population, that is the Lifelines population. We estimated these prevalences using plug-in methods from the “marginaleffects” package in R (Arel-Bundock, Greifer, & Heiss, 2024). First, we fitted a logistic regression model in which disease status (case or control) was regressed on whether a person had an affected relative while adjusting for age, age^2^, sex, and number of relatives in the data. This model quantified how these factors are associated with disease risk. Second, we estimated disease prevalences by plugging-in the fitted model to our study population: once with their existing covariate values to estimate prevalence in the general population, and once with each participant’s relative affected status set to ‘true’ (while keeping existing values for age, sex, and family size) to estimate prevalence among those with affected relatives. The ratio of these two prevalence estimates gives the marginal λ_R_, which represents how much having an affected relative increases disease risk, averaged across the total population’s distribution of age, sex, and family size. See supplementary methods for details. To ensure that we capture the full complexity of familial relationships without artificially separating comorbid cases, individuals with multiple diagnoses were included in the analysis for each of their conditions. To minimize reporting bias, proband reports of relatives’ disease status were not used (Milne et al., 2009; Saito et al., 2008). To account for correlated observations due to familial clustering, 95% confidence intervals (CIs) were estimated using a robust clustered sandwich method (Zeileis, Köll, & Graham, 2020). In addition to calculating the λ_R_ for first-degree relatives in general, λ_R_ was also calculated for siblings, parents, and offspring separately and for second-degree relatives. Furthermore, λ_R_ was calculated for cohabiting spouses as a measure of spousal resemblance, which can arise as the result of assortative mating and/or shared environmental influences.

Unlike λ_R_, tetrachoric correlations are relatively insensitive to differences in the prevalence of the disorders involved in the calculations (Babchishin & Helmus, 2016; Cummings, 2009). Therefore, tetrachoric correlations were calculated for all parent-offspring pairs/trios and sibling pairs within families as an additional measures of familial aggregation and coaggregation. These correlations accounted for nonindependence within nuclear families within MPlus (Muthén & Muthén, 1998).

#### Familiality and familial correlation

The terms heritability and genetic correlation imply that familial resemblance is solely due to genetics. Unlike twin studies, our family study did not disentangle genetic from shared environmental influences because an estimation of shared environment based on, for example, a comparison of first- and second-degree relatives would have limited power. To address this conflation and the resulting incomparability to heritability estimates from twin studies, we use the terms familiality and familial correlation instead of heritability and genetic correlation (Kendler & Neale, 2009).

To estimate the familiality of and familial correlations between FDs and IDs, we used the methods of Falconer (1965) and Reich, James, and Morris (1972), as adapted by Wray and Gottesman (2012) (Baselmans, Yengo, van Rheenen, & Wray, 2021). These methods are based on the liability threshold model, which assumes that a normally distributed liability underlies disease status. Individuals exceeding the critical liability threshold are affected. Although unobservable, this threshold can be determined using normal distribution theory, given the proportion of affected individuals in the population. The prevalence of disease in the general population and in thefirst- and second-degree relatives of affected individuals was used to estimate both the familiality of the disorders and the familial correlations between disorders. See supplementary methods for details.

#### Sensitivity analyses

We performed three sensitivity analyses. First, given the functional limitations associated with IDs and FDs (Buist-Bouwman et al., 2006; Joustra et al., 2015), we hypothesized a higher dropout rate between assessment waves for individuals with affected relatives. This selective dropout could lead to underestimating disease prevalence in relatives of affected individuals, consequently resulting in an underestimation of λ_R_ and familiality. We used logistic regression to compare participation in the second and third waves between participants with and without affected first-degree relatives in wave 1 or 2, adjusting for age, sex, and the number of participating relatives in wave 2. For IDs, the participant’s disease status in wave 1 was also considered, which was not possible for FDs as they were not assessed in wave 1. For wave 3, we additionally evaluated if individuals with affected relatives in any of the three waves were less likely to participate. Models were adjusted for age, sex, number of participating relatives in wave 3, and the individual’s disease status in previous waves.

Second, to ensure specificity to FDs, participants who met the diagnostic criteria for an FD and reported a medical condition with similar symptoms were excluded from the familial coaggregation analyses. For ME/CFS, participants with multiple sclerosis (MS), dementia, schizophrenia, or an eating disorder were excluded. For IBS, participants with ulcerative colitis, Crohn’s disease, or coeliac disease were excluded. For FM, participants with rheumatoid arthritis were excluded. Additionally, hepatitis, cancer, or heart failure were exclusion criteria for all FDs.

Third, we assessed the impact of differences in diagnostic criteria strictness for FDs. The diagnostic criteria for ME/CFS are stricter than those for IBS and FM; ME/CFS requires interference with daily tasks, while IBS and FM do not. Moreover, ME/CFS and IBS require a six-month duration, in contrast to three months for FM. Familial coaggregation is often stronger for more severe phenotypes (Steinhausen, Jakobsen, & Munk-Jørgensen, 2017; Wang, Snieder, & Hartman, 2022). Therefore, the duration and interference criteria of FM and IBS were aligned with ME/CFS criteria, meaning that a symptom duration of six months and interference with daily life activities were required for all three FDs. This helped assess to which extent differences between FDs in λ_R_ and tetrachoric correlations were due to (arbitrary) diagnostic criteria or due to a difference in the type of symptoms.

#### Reporting and software

Results are considered significant if p < .01. Mplus version 8.2 (Muthén & Muthén, 1998) was used to calculate tetrachoric correlations. R Version 4.2.1 (R Core Team, 2022) was used for all other analyses. R scripts are available at Open Science Framework (OSF), via doi: 10.17605/OSF.IO/7RCVT

## Results

### Descriptives

The diagnostic status for IDs or FDs could be determined for 153,803 Lifelines participants, based on up to three assessments between 2006 and 2023 for IDs and up to two assessments between 2014 and 2023 for FDs. The descriptive characteristics of these individuals are presented in Table 1. For 90,397 (58.8%) individuals, it was possible to determine disease status for at least one first-degree relative. The disease status of a second-degree relative could be assessed for 23,978 (15.6%) individuals. The data included 37,184 sibling pairs from 23,185 nuclear families, 16,455 parent-offspring pairs (a child with only one parent) from 11,916 nuclear families, and 17,046 parent-offspring trios (a child with both parents) from 11,357 nuclear families.

**Table 1.** Descriptive characteristics of dataset

	N (total dataset/subjects with first-degree relative in dataset)	Total(N = 153,803)	Participants with first-degree relatives in data (N = 90,397)
Demographics			
Age^a^, mean (SD)		44.2 (13.4)	44.4 (14.9)
Sex (Female), %		58.6	60.1
Parent in dataset, %		21.8	37.1
Sibling in data, %		34.1	58.0
Child in data, %		22.4	38.1
Spouse^b^ in data, %		38.9	41.7
Internalizing disorders^c^			
MDD, %	152,633/89,614	4.8	4.3
DYS^d^, %	150,462/88,543	2.4	2.2
GAD, %	152,629/89,614	9.1	8.4
AGPH, %	152,629/89,615	5.8	5.7
SPH, %	152,626/89,614	1.9	1.8
PD, %	152,627/89,614	1.2	1.1
Functional disorders^e^			
ME/CFS, %	102,648/63,185	4.7	4.4
IBS, %	102,847/63,285	7.5	7.5
FM^f^, %	97,378/59,983	8.2	7.7

Abbreviations: SD, standard deviation; MDD, major depressive disorder; DYS, dysthymia; GAD, generalized anxiety disorder; AGPH, agoraphobia; SPH, social phobia; PD, panic disorder; ME/CFS, myalgic encephalomyelitis/chronic fatigue syndrome; IBS, irritable bowel syndrome; FM, fibromyalgia.

a Age during the first adult assessment an individual participated in.

b Pairs were considered spouses if they lived together during the baseline assessment and indicated to be spouses or share a child together. This includes same-sex spouses.

c Cases aggregated across assessment waves 1, 2, and 3. Lifetime diagnoses were not established.

d Sample size of DYS is smaller than for other IDs as in some assessment waves DYS questions were skipped if MDD was present.

e Cases aggregated across assessment waves 2 and 3. Functional disorders were not assessed in first assessment wave. Lifetime diagnoses were not established.

f Sample size of FM is smaller than for other FDs as FM questions were spread across two questionnaires.

### Familial aggregation and coaggregation

**Figure 1.**
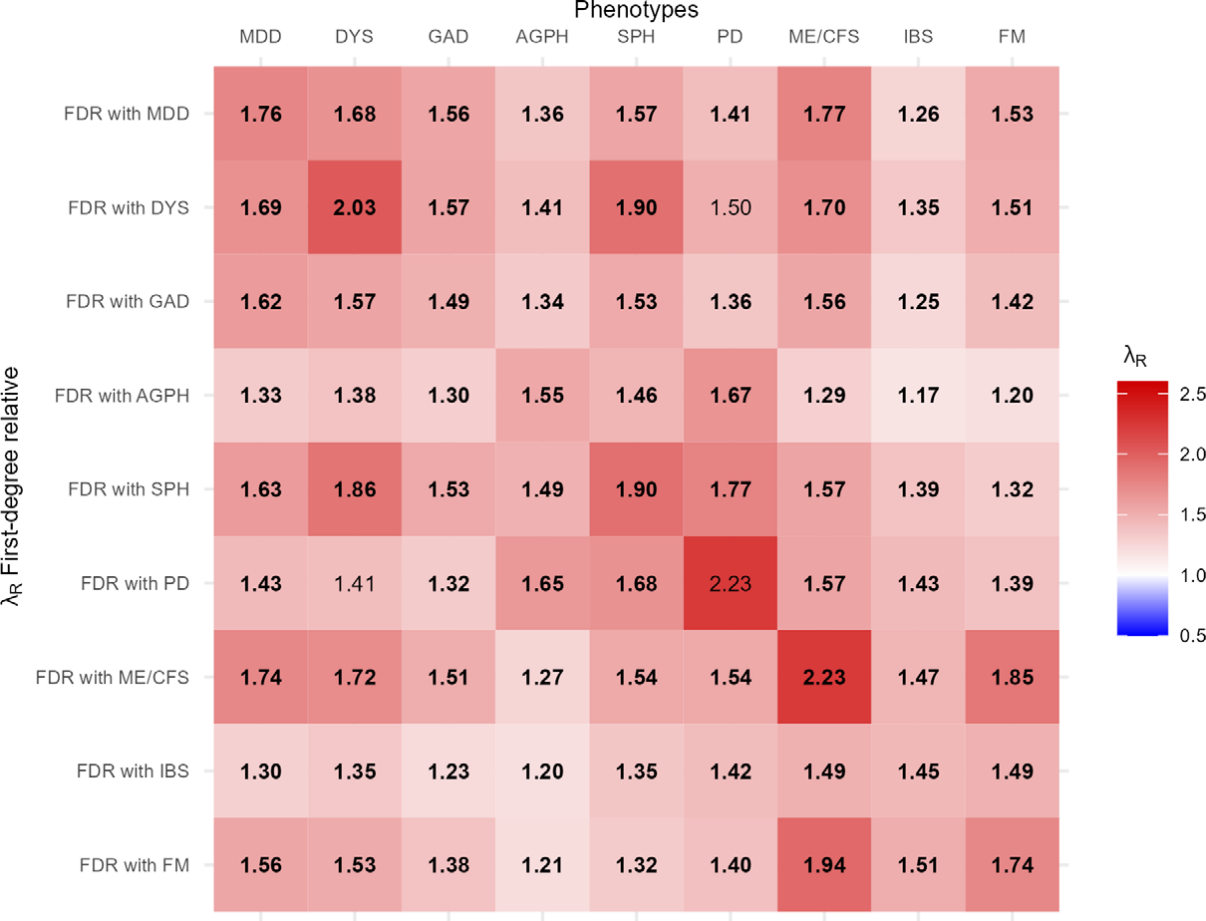
Familial coaggregation of internalizing and functional disorders amongst first-degree relatives expressed in λ_R_. *Note:* FDR, first-degree relative; MDD, major depressive disorder; DYS, dysthymia; GAD, generalized anxiety disorder; AGPH, agoraphobia; SPH, social phobia; PD, panic disorder; ME/CFS, myalgic encephalomyelitis/chronic fatigue syndrome; IBS, irritable bowel syndrome; FM, fibromyalgia; λ_R_, recurrence risk ratios. λ_R_ adjusted for age, age^2^, sex, and number of relatives present in the data. Estimates in bold are significant at p < .01. For 95% confidence intervals, see Supplementary Table 1.

For all disorders except PD and DYS, having a first-degree relative affected by the disorder was significantly associated with a higher risk for all of the other disorders (Figure 1). Estimates of λ_R_ ranged from 1.17 (95% CI: 1.06–1.27) for IBS with a relative affected by AGPH to 2.23 for both PD and ME/CFS with a relative affected by the same disorder (95% CI: 1.26–3.20 for PD, 1.89–2.58 for ME/CFS). Familial coaggregation within IDs was similar to that within FDs. Among FDs, ME/CFS showed the strongest coaggregation with IDs, while IBS showed the weakest. Generally, familial coaggregation did not exhibit different patterns by type of first-degree relative (Supplementary Table 1).

**Figure 2.**
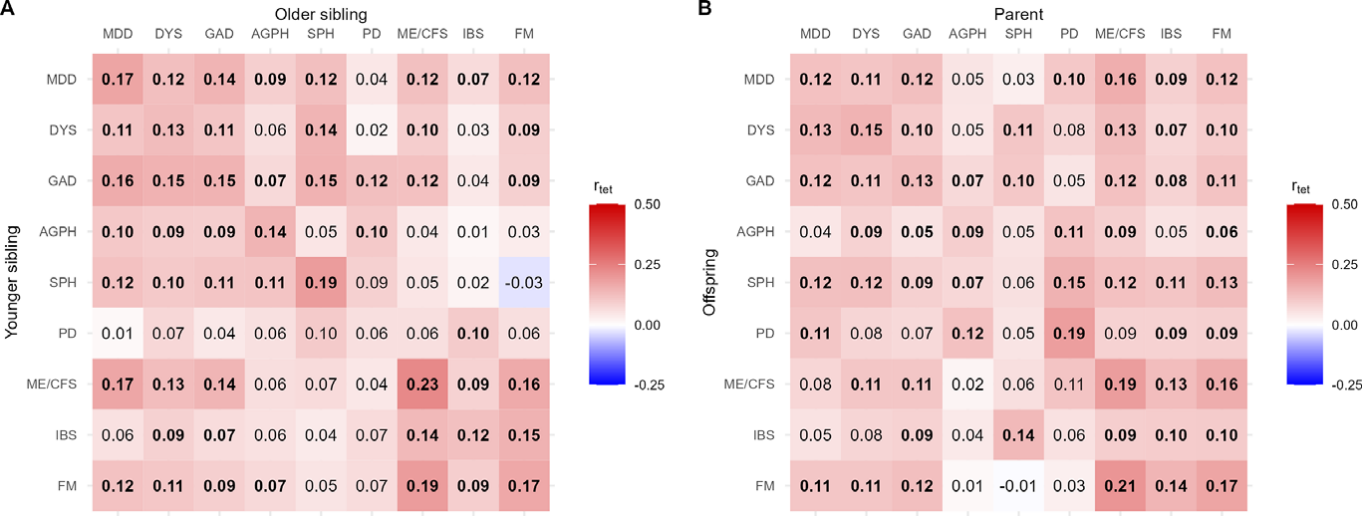
Tetrachoric correlations between internalizing and functional disorders, for (a) siblings and (b) parent-offspring pairs/trios. *Note:* MDD, major depressive disorder; DYS, dysthymia; GAD, generalized anxiety disorder; AGPH, agoraphobia; SPH, social phobia; PD, panic disorder; ME/CFS, myalgic encephalomyelitis/chronic fatigue syndrome; IBS, irritable bowel syndrome; FM, fibromyalgia; r_tet_, tetrachoric correlation. Estimates in bold are significant at p < .01. For 95% confidence intervals, see Supplementary Tables 2 & 3.

Unlike the λ_R_ estimates for all first-degree relatives combined, tetrachoric correlations calculated between sibling pairs and between parent-offspring pairs/trios did not consistently reach statistical significance (Figure 2). Patterns of familial coaggregation expressed in tetrachoric correlations did align with λ_R_ of sibling and parents-offspring pairs (Supplementary Tables 1–3). For instance, IBS was less strongly associated with IDs than ME/CFS and FM.

**Figure 3.**
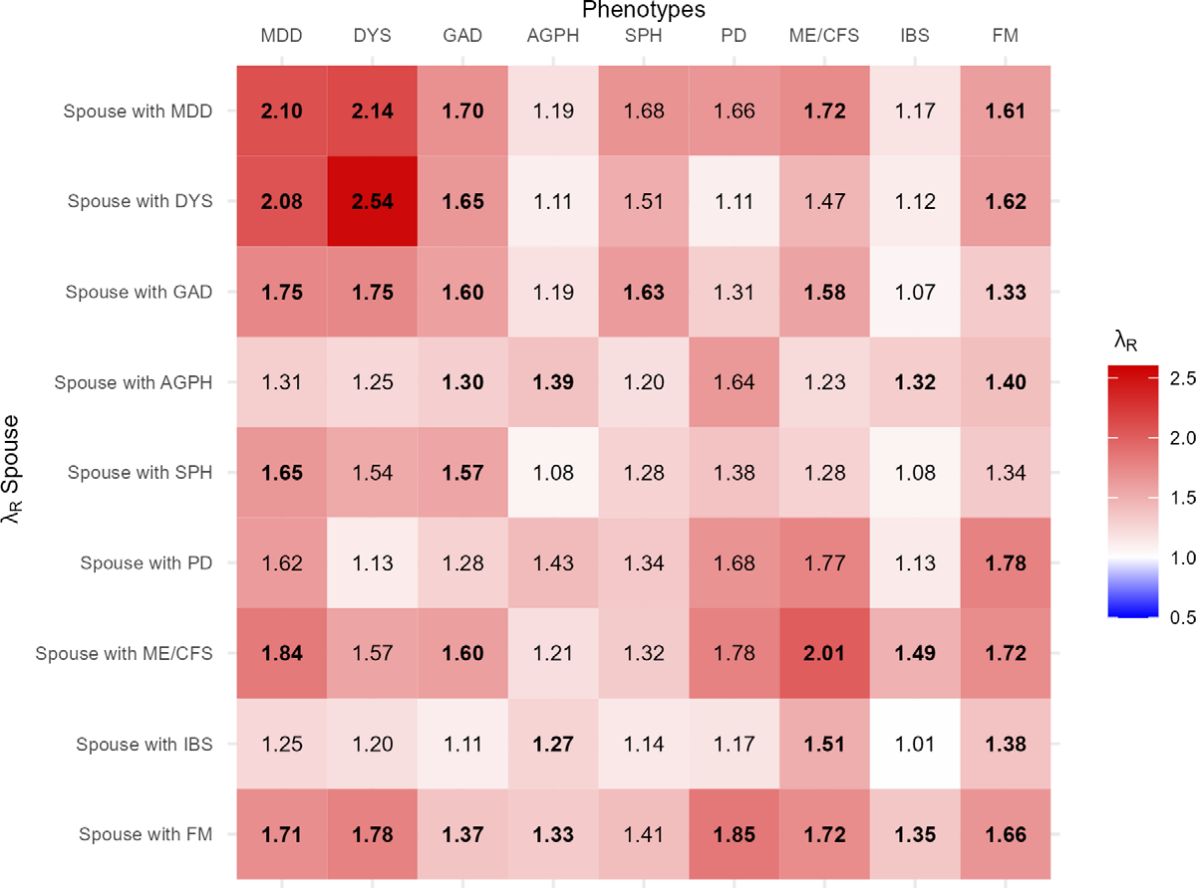
Familial coaggregation of internalizing and functional disorders amongst spouses, expressed in λ_R_. *Note:* MDD, major depressive disorder; DYS, dysthymia; GAD, generalized anxiety disorder; AGPH, agoraphobia; SPH, social phobia; PD, panic disorder; ME/CFS, myalgic encephalomyelitis/chronic fatigue syndrome; IBS, irritable bowel syndrome; FM, fibromyalgia; λ_R_, recurrence risk ratio. λ_R_ adjusted for age, age^2^, sex, and presence of spouse in the data. Estimates in bold are significant at p < .01. For 95% confidence intervals, see Supplementary Table 1.

Within disorders, significant spousal resemblance was observed for MDD, DYS, GAD, AGPH, ME/CFS, and FM. Furthermore, all disorders showed spousal coaggregation with at least one other disorder (Figure 3). In some cases, λ_R_ for spouses was larger than λ_R_ for first-degree relatives. For instance, for MDD λ_R_ for first-degree relatives was 1.76 (95% CI: 1.54–1.98) while λ_R_ for spouses was 2.10 (95% CI: 1.63–2.56).

### Familiality and familial correlation

**Figure 4:**
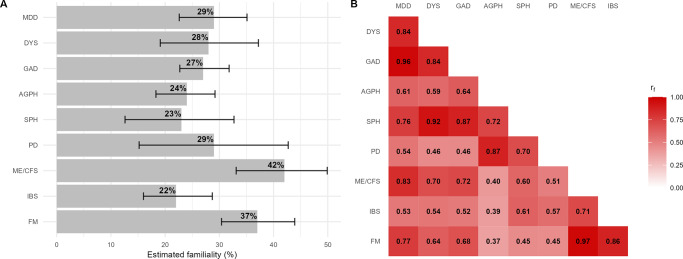
(a) Familiality and (b) familial correlation estimates of internalizing and functional disorders based on both first- and second-degree relatives. *Note:* MDD, major depressive disorder; DYS, dysthymia; GAD, generalized anxiety disorder; AGPH, agoraphobia; SPH, social phobia; PD, panic disorder; ME/CFS, myalgic encephalomyelitis/chronic fatigue syndrome; IBS, irritable bowel syndrome; FM, fibromyalgia; r_f_, familial correlation. See Supplementary Table 4 for familiality estimates for first- and second-degree relatives separately and Supplementary Table 5 for 95% confidence intervals of familial correlation estimates.

In general, familiality estimates were modest to moderate, ranging from 22% (95% CI: 16–29) for IBS to 42% (95% CI: 33–50) for ME/CFS (Figure 4a). Familiality estimates based on second-degree relatives were similar to those based on first-degree relatives, except for SPH and ME/CFS (Supplementary Table 4). This suggests limited influences from shared environment for most disorders as second-degree relatives typically share much less of their family and community environment than do first-degree relatives. Moderate to high familial correlations were observed across disorders, ranging from +0.37 (95% CI: 0.24–0.51) between AGPH and FM to +0.97 (95% CI 0.80–1) between ME/CFS and FM. The familial correlation between MDD and ME/CFS was +0.83 (95% CI: 0.66–0.99), which was the strongest familial correlation observed between an ID and FD (Figure 4b).

### Sensitivity analysis

We assessed whether dropout between waves was higher among participants whose first-degree relative was affected by any of the disorders, as this could lead to an underestimation of disease prevalence in relatives of affected individuals. Individuals with a first-degree relative affected by MDD, GAD, AGPH, PD, or FM during the first or second assessment waves were less likely to participate in the MINI of wave 2. Those with a first-degree relative affected by PD were least likely to participate (OR: 0.86, 95% CI: 0.77–0.97). Individuals with a first-degree relative affected by GAD in wave 1 or 2 were also less likely to participate in the FD questionnaire of wave 2 (OR: 0.90, 95% CI: 0.86–0.94). For wave 3, individuals with a first-degree relative affected by MDD, GAD, AGPH, CFS, or FM in any of the assessment waves were less likely to participate in both the MINI and the FD questionnaire. Individuals whose first-degree relative was affected by FM were least likely to participate in the wave 3 MINI (OR: 0.86, 95% CI: 0.81–0.91) and the FD questionnaire (OR: 0.87, 95% CI: 0.81–0.92). See Supplementary Table 6 for all logistic regression results.

To ensure specificity to FDs, subjects with somatic disorders were excluded from familial aggregation and coaggregation analyses, reducing the prevalence of ME/CFS, IBS, and FM to 3.9%, 6.3%, and 6.6%, respectively. This exclusion had no to a minimal impact on familial coaggregation estimates (Supplementary Tables 7–9).

To account for differences in strictness of diagnostic criteria, IBS and FM duration and interference criteria were aligned with ME/CFS to include 6-6-month duration and interference with daily activities. This reduced IBS prevalence from 7.5% to 1.1% and FM prevalence from 8.2% to 4.5%. Severity-aligned IBS coaggregated more with IDs than the original criteria. Although ME/CFS still showed stronger coaggregation with IDs than severity-aligned FM, differences did attenuate, indicating that criteria strictness partly explains the familial aggregation differences (Supplementary Tables 10–12).

## Discussion

This study assessed the familial aggregation and coaggregation, as well as the familiality of and familial correlations between FDs and IDs in a large general population sample. We observed significant aggregation within and between studied disorders. Furthermore, each disorder showed spousal coaggregation with at least one other disorder. Whether this results from assortative mating or shared environment is unknown. We also observed moderate familiality across all disorders, with familial correlations indicating that this familiality is moderately to highly shared between FDs and IDs.

### Comparison with familial coaggregation studies

Our results show that individuals with a first-degree relative affected by an ID or an FD are at increased risk of developing the same or other studied disorders, consistent with previous studies on IDs (Hettema, Neale, & Kendler, 2001; Sullivan, Neale, & Kendler, 2000), FDs (Albright et al., 2011; Saito et al., 2010) and across IDs and FDs (Allen-Brady et al., 2023; Hudson et al., 2004). Our coaggregation patterns largely resemble those of a medical records-based study in a population registry database (Allen-Brady et al., 2023). Minor discrepancies, like IBS exhibiting stronger familial aggregation than FM in the previous study (Allen-Brady et al., 2023) might be due to the severity of IBS cases in medical records. Our sensitivity analysis supports this explanation, as severe IBS that interferes with daily activities showed stronger familial aggregation than noninterfering IBS. A study focusing on FM mirrored our findings with MDD but reported no significant coaggregation with GAD, SPH, PD, or IBS, possibly due to limited sample size and the small number of individuals affected by these disorders (Hudson et al., 2004). In contrast to λ_R_ in all first-degree relatives, tetrachoric correlations between sibling and parent-offspring pairs in the current study were not always significant, likely due to reduced statistical power and methodological differences. For instance, unlike λ_R_, tetrachoric correlations assume an underlying normally distributed liability to disease. Although this is a common assumption in genetic epidemiology, it may not fully reflect the complex familiality of these disorders.

### Comparison with genetic studies

We use the terms familiality and familial correlation rather than heritability and genetic correlation because our methods do not disentangle genetic from shared environmental effects. However, evidence suggests that for most disorders, familial aggregation results from genetic factors, with limited impact from shared environmental factors. For instance, in twin studies on IDs, IBS, and FM, models not including a shared environmental component are a better fit for the data than models that do include a shared environmental component (Hettema et al., 2001; Magnusson et al., 2024; Markkula et al., 2009; Sullivan et al., 2000; Svedberg et al., 2008). Additionally, except for SPH and ME/CFS, our familiality estimates from second-degree relatives were very close to those from first-degree relatives (Supplementary Table 4), despite second-degree relatives sharing less of their environment than first-degree relatives. This also suggests a limited role of the shared environment. Moreover, for well-studied disorders like MDD and GAD, our familiality estimates are lower than the heritability estimates from meta-analyses of twin studies (Hettema et al., 2001; Sullivan et al., 2000), which would be unlikely if there were strong shared environmental effects. The familial correlation between MDD and GAD also aligns with the genetic correlation found in a major twin study (Kendler, Gardner, Gatz, & Pedersen, 2007). Therefore, we believe that the current study contributes to the growing body of evidence supporting a genetic component in FDs (Ablin & Buskila, 2015; Dibble, McGrath, & Ponting, 2020; Saito, 2011).

Our familiality estimates for ME/CFS and IBS are similar to heritability estimates from twin studies (Buchwald et al., 2001; Svedberg et al., 2008). Our familiality estimate for FM is higher than the heritability estimate (23%, 95% CI 14–32) of a twin study based on ICD-10 codes for myalgia (muscle pain) and FM (Magnusson et al., 2024). In contrast, our estimate is lower than the estimate (51%, 95% CI: 45–56) of a twin study that identified FM through questionnaire items related to symptoms like morning and evening stiffness, neck pain and stiffness, tender points, daytime fatigue, and numbness (Markkula et al., 2009).

Our findings also suggest that the liability to disease is shared between FDs and IDs, corroborating previous studies (Allen-Brady et al., 2023; Kato et al., 2009; Kendler et al., 2023). A Swedish twin study identified two latent genetic variables that underlie the comorbidity of MDD, GAD, and FDs (Kato et al., 2009). MDD, GAD, and FDs loaded on one latent genetic variable, which is in line with the familial correlations we found between IDs and FDs. The second latent genetic variable was exclusively related to FDs (Kato et al., 2009), fitting the high familial correlations we found between FDs. Strong correlations between FDs could be because individuals meeting criteria for one FD often report symptoms included in the diagnostic criteria for other FDs (van der Meulen et al., 2024).

ME/CFS and FM exhibited a closer familial relationship than either had with IBS, while IDs were more linked to ME/CFS than to FM or IBS. These findings parallel the factor loadings of the Swedish twin study (Kato et al., 2009). However, they differ from a family-based Swedish registry study, where FM and ME/CFS were more related to IBS than to each other, and IDs were most associated with FM (Kendler et al., 2023). Similarly, a medical records-based study found that IBS, FM, and MDD showed more extensive overlap with other FDs or MDD in distant relatives than ME/CFS (Allen-Brady et al., 2023). These differences could stem from diagnostic biases (Tattan et al., 2024) and overrepresentation of severe cases in registry/records-based studies, unlike the self-reported symptom assessments in our general population study and the Swedish twin study (Kato et al., 2009).

### Relevance and future research

The observed familial coaggregation and familial correlations between FDs and IDs suggest shared etiological mechanisms between these groups of disorders. The specific shared mechanisms remain unknown and most likely reflect multilevel, interlocking etiological pathways (Kendler, 2012). For instance, emotion regulation problems have been linked to IDs, ME/CFS, and FM (Bram, Gottschalk, & Leeds, 2018; Picó-Pérez et al., 2017; Pinto et al., 2023), while inflammation has been associated with FDs and MDD (Andrés-Rodríguez et al., 2020; Burns et al., 2019; Harsanyi, Kupcova, Danisovic, & Klein, 2023; Strawbridge, Sartor, Scott, & Cleare, 2019). Genetic overlap previously found between FDs and somatic disorders further emphasizes the multifaceted nature of FD etiology (Kendler et al., 2023).

To uncover specific shared biological mechanisms, molecular genetic studies are needed. A GWAS revealed shared loci between IBS and anxiety disorders (Eijsbouts et al., 2021). Genes mapped to shared loci between IBS and anxiety disorders regulate neural circuits and influence white matter microstructure (Eijsbouts et al., 2021) and were enriched for pathways relevant to the nervous and immune system (Tesfaye et al., 2023). While our findings advance the understanding of FDs, extensive molecular genetic studies, including ME/CFS and FM, are needed to identify shared genomic loci, which could unveil the underlying mechanisms common to IDs and FDs.

Genetic correlations may also stem from a causal link on the symptom level. For instance, sleep dysfunction, which is a symptom of MDD, is a potential trigger for FM (Choy, 2015). Mendelian randomization studies could provide additional understanding of the causal relationship between FDs and IDs (Davey Smith & Ebrahim, 2004).

### Strengths and limitations

This study’s strengths lie in its use of a large population-based cohort and a diagnostic algorithm for assessing self-reported symptoms against official diagnostic criteria. By avoiding registry data, medical center recruitment, or self-reported diagnoses, we minimized the impact of diagnostic biases and help-seeking behavior (Kendler, 1995; Talley & Spiller, 2002; Tattan et al., 2024; Wolfe et al., 2019), prevented oversampling of severe cases, and captured cases otherwise being overlooked (Warren & Clauw, 2012). Furthermore, the direct assessment of disease status in relatives minimizes potential biases associated with participants reporting on the health of their relatives, addressing concerns related to incomplete knowledge about family members’ health (Milne et al., 2009; Saito et al., 2008).

The primary limitation of this study is the potential inaccuracy in estimating disease prevalence among individuals with an affected relative. This arises from several factors. Firstly, we lacked complete data on first-degree relatives for all Lifelines participants, with 41% of participants having no first-degree relatives in the data. These individuals could not be considered for the population of individuals with an affected relative. By including these individuals in the general population estimate, we implicitly assumed they were representative of the general population. However, our data suggest that this might not be the case as disease prevalence was lower among those with relatives in the dataset (Table 1), suggesting that individuals without relatives were less healthy. Recognizing that the number of family members in the data is associated with both our outcome (disease prevalence) and our exposure (having an affected relative), we included the number of relatives in the data as a covariate in our analyses, addressing some of the inaccuracies in prevalence estimation. However, a second limitation is participation bias, as individuals with relatives affected by MDD, GAD, AGPH, PD, IBS, or FM were less likely to participate in subsequent assessment waves. This likely resulted in an underestimation of disease prevalence among those with affected relatives. Moreover, in the absence of lifetime diagnoses, we relied on point-in-time diagnoses. These limitations have possibly led to an underestimation of λ_R_ and familiality estimates.

## Conclusion

In conclusion, our study underscores familial and likely genetic overlap between FDs and IDs, suggesting potential shared etiological mechanisms. To clarify the specific nature of these shared mechanisms and explore potential causal relationships between FDs and IDs, additional studies incorporating genotype data are needed.

## Supporting information

Bos et al. supplementary material 1 Bos et al. supplementary material

Bos et al. supplementary material 2 Bos et al. supplementary material

## Data Availability

Code for estimating λ_R_, familiality, and familial correlations are available on OSF via doi: 10.17605/OSF.IO/7RCVT.

## Supplementary material

The supplementary materials for this article can be found at https://doi.org/10.1017/S003329172500100X.
