# Supplementary material for: Familial coaggregation and shared familiality of functional and internalizing disorders in the Lifelines cohort
Source: Psychol Med. 2025 May 2;55:e126. doi: 10.1017/S003329172500100X (PMC12094627; doi:10.1017/S003329172500100X)
Supplement: Bos et al. supplementary material 1 — Bos et al. supplementary material [file S003329172500100Xsup001.docx]

**Supplementary methods of Familial co-aggregation and shared familiality of functional and internalizing disorders in the Lifelines cohort**

Martje Bos, MPhil^1,*^,

Rei Monden, PhD^1,4^,

Naomi R. Wray, PhD^5,6^,

Yiling Zhou, MSc^3^,

Kenneth S. Kendler, MD/PhD^7,8^

Judith G. M. Rosmalen^1,2^, PhD^1,2,✝^,

Hanna M. van Loo, MD/PhD^1,✝^,

Harold Snieder, PhD^3,✝^

Affiliations:
Departments of ^1^Psychiatry, ^2^Internal Medicine, and ^3^Epidemiology, University of Groningen, University Medical Center Groningen, Groningen, the Netherlands.

^4^Informatics and Data Science Program, Graduate School of Advanced Science and Engineering, Hiroshima University, Higashi-Hiroshima, Hiroshima, Japan

^5^ Institute for Molecular Bioscience, The University of Queensland, Brisbane, QLD, Australia

^6^ Department of Psychiatry and Big Data Institute, University of Oxford, Oxford, United Kingdom

^7^ Virginia Institute for Psychiatric and Behavioral Genetics, Virginia Commonwealth University, Richmond, VA, USA.

^8^ Department of Psychiatry, Virginia Commonwealth University, Richmond, VA, USA

^✝^Authors supervised this work

*** Address correspondence to**: M. Bos, University of Groningen, University Medical Center Groningen, Psychiatry, Hanzeplein 1, 9700 RB Groningen, The Netherlands; Email: [m.bos03@umcg.nl](mailto:m.bos03@umcg.nl)


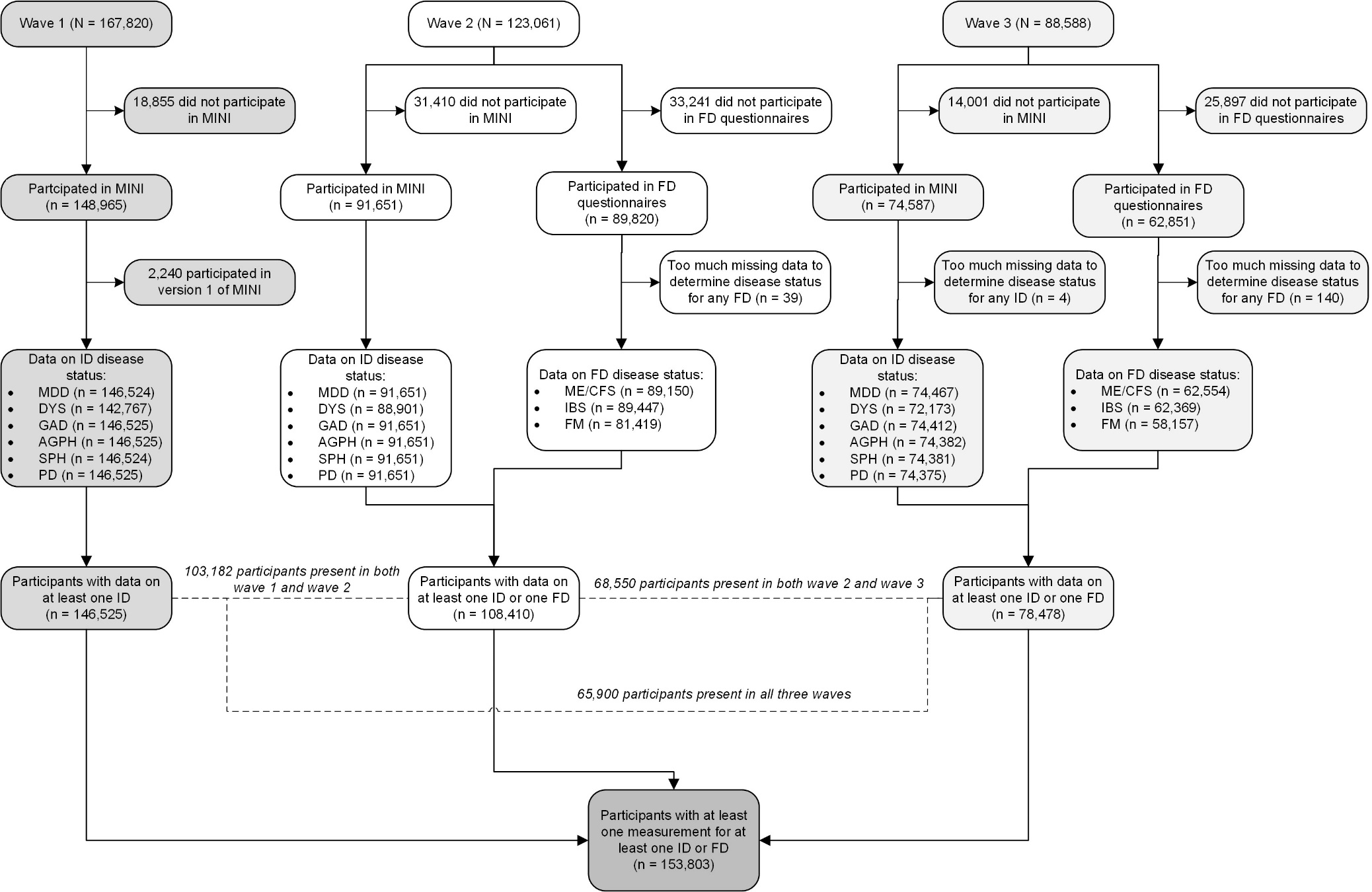
**Supplementary Table 1: Flowchart of how data of 153,803 adults participants with at least one measurement on IDs or FDs was included in this study.**  Total N per wave includes all Lifelines participants who participated in at least one measurement in that particular wave, including participants younger than 18 years of age. MINI, Mini-International Neuropsychiatric Interview; FD, functional disorder; ID, internalizing disorder; MDD, major depressive disorder; DYS, dysthymia; GAD, generalized anxiety disorder; AGPH, agoraphobia; SPH, social phobia; PD, panic disorder; ME/CFS, myalgic encephalomyelitis/chronic fatigue syndrome; IBS, irritable bowel syndrome; FM, fibromyalgia.

**Methods**

Measurements

*Internalizing disorders*

In all three assessment waves of the Lifelines cohort study, current major depressive disorder (MDD), dysthymia (DYS), generalized anxiety disorder (GAD), agoraphobia (AGPH), social phobia (SPH), and panic disorder (PD) were evaluated using the Mini-International Neuropsychiatric Interview (MINI) (Sheehan et al., 1998), which assesses the symptoms of these six internalizing disorders (IDs) according to DSM-IV criteria (American Psychiatric Association, 2000). Across the three assessment waves of the Lifelines cohort, five versions of the MINI have been used. Version 1 was not used in the current study, as this assessed IDs in the past year. Versions 2-5 of the MINI assessed the presence of MDD, DYS, and GAD in the past two weeks, two years, and six months, respectively. The presence of AGPH, SPH, and PD was assessed in the past month (van Loo et al., 2023).

During the baseline assessment of Lifelines, versions 1, 2, and 3 of the MINI were administered as a face-to-face interview by a trained research nurse at a Lifelines research facility. In the second assessment, participants completed a digital MINI questionnaire (MINI version 4) at the research facility. In these two assessments, questions related to DYS were skipped if a participant met diagnostic criteria for MDD (MINI versions 3 and 4) or if least one core criterion and three additional criteria of MDD were present (MINI version 2). For the third assessment, a digital MINI questionnaire (MINI version 5) was sent to participants’ email so that they could complete the MINI at home. In MINI version 5, questions relating to DYS were skipped if the core criteria of MDD were not present. This contrasts the skipping pattern of MINI versions 2-4 and resulted in a large amount of missing data for DYS in assessment wave 3. To address this issue, we decide to change a missing DYS diagnosis to a control if all of the following three condition were met: 1) all DYS items were missing; 2) the participant reported that they did not have a depressed mood or lost interest in activities in the past two weeks; 3) the participant reported that they never had a two week period of a depressed mood or lost interest in activities in their life.

For this study, ID data from all three assessment waves was used. Participants were coded a control for a disorder if they did not meet diagnostic criteria for that disorder in any of the assessment waves they participated in. Participants were coded as a case if they met diagnostic criteria in at least one of the assessment waves they participated in. If too little data was available to reliably determine whether a participant met the diagnostic criteria, their diagnostic status was coded as missing.

*Functional disorders*

In the second and third assessment of Lifelines, chronic fatigue syndrome (ME/CFS), fibromyalgia (FM) and irritable bowel syndrome (IBS) were assessed to official diagnostic criteria. Supplementary Table 1 of previous work describes in detail how these official diagnostic criteria were applied in Lifelines (van Loo, Maeder, Bos, & Rosmalen, Manuscript under review). Participants were coded a control for a disorder if they did not meet diagnostic criteria for that disorder in any of the assessment waves they participated in. Participants were coded as a case if they met diagnostic criteria in at least one of the assessment waves they participated in. If too little data was available to reliably determine whether a participant met the diagnostic criteria, their diagnostic status was coded as missing.

Statistical analyses

*Recurrence risk ratios*

Recurrence risk ratios (λ_R_s) were calculated to assess familial aggregation and co-aggregation (Risch, 1990). $\lambda_{R}$ was defined as the ratio between the prevalence in relatives of participants affected by the disorder under study and the prevalence in the general population, i.e. the Lifelines population:

$\lambda_{R}= \frac{K_{r}}{K}$ Equation 1

Here, $K$ refers to the prevalence of the disorder in the general Lifelines population, and $K_{r}$ is the prevalence in relatives of affected individuals.

We estimated marginal $K$ and marginal $K_{r}$ using a two-phase process. In the first phase, the disease probability was modeled using logistic regression

$\log\left( \frac{Pr(D=1)}{1-Pr(D=1)} \right)= \beta_{0}+ \beta_{1}X_{1}+ \beta_{2}X_{2}+ \beta_{3}X_{3}+ \beta_{4}X_{4}+\beta_{5}X_{5}$ Equation 2

where $D$ = disease status (1 for cases, 0 for controls); $X_{1}$= age; $X_{2}$= age^2^; $X_{3}$= sex; $X_{4}$= number of relatives in the dataset; and $X_{5}$= affected relative status (1 if at least one relative is affected, 0 if no relative is affected).

In the second phase, marginal $K$ and marginal $K_{r}$ were estimated using the avg_predictions() function from the "marginaleffects" package for R (Arel-Bundock, 2024). The avg_predictions() function estimates the prevalence of $D=1$given the combination of values of the covariates in the logistic regression model. For $K$, this function was applied to the entire dataset using the existing covariate values. For $K_{r}$, the same function was applied under a simulated scenario where $X_{5}$was set to 1 for all observations.

Importantly, the resulting $K$ and $K_{r}$ estimates share the same covariate distribution, and therefore the ratio of $K_{r}$ to $K$ gives a marginal $\lambda_{R}$ over the distribution of age, age^2^, sex, and number of relatives in the total population. The adjustment for the number of relatives is important as participants without any relatives in the dataset will automatically have no affected relatives, while those with many relatives are more likely to have an affected relative merely due to chance.

**Formulas for familiality and familial correlation estimation**

In the liability threshold model, individuals are considered diseased if their phenotypic liability (Z ∼ N(0,1)) exceeds threshold $T$, making the distribution $p\left( Z>T \right)=K$. For relatives of affected individuals, the threshold for disease is $T_{R}$, so the distribution is $p\left( Z>T_{R} \right)=K_{r}$ (Falconer, 1965; Reich, James, & Morris, 1972; Wray & Gottesman, 2012).

We calculated familiality ($f^{2}$) of disease and its approximate standard error (s.e.) based on the model-derived $K$ and $K_{r}$ from the $\lambda_{R}$ estimates as follows:

$f^{2}=\frac{T-T_{R}\times\sqrt{1-(1-\frac{T}{i})\times(T^{2}-T_{R}^{2})}}{a_{R}\times(i+(i-T)\times T_{R}^{2})}$ Equation 3

$s.e\left( f^{2} \right)\approx\frac{1}{a_{R}}\times\sqrt{[\frac{{s.e(K)}^{2}}{y^{2}}\times\left( \frac{1}{i}+a_{R}\times f^{2}\times\left( i-T \right) \right)^{2}+\frac{{s.e(K_{R})}^{2}}{i^{2}\times y_{R}^{2}}}]$ Equation 4

Here, $i$ is is the mean liability of the diseased group in the population, calculated as $i=\frac{y}{K}$  where $y$ is the height of the normal curve at threshold $T$. $a_{R}$ is the additive genetic relationship between relatives. We calculated $f^{2}$ based on first-degree relatives ($a_{R}$ = 0.5) and second-degree relatives ($a_{R}$ = 0.25). We obtained overall $f^{2}$ estimates by weighting the first- and second-degree relative estimates by the inverse of their sampling variances.

To calculate familial correlations between disorders b and c, we first calculated their co-familiality as described in equations 5 and 6

$r_{bc}f_{b}f_{c}=\frac{T_{b}-T_{bc}\sqrt{1-(1-\frac{T_{c}}{i_{c}})(T_{b}^{2}-T_{bc}^{2})}}{a_{R}(i_{c}+\left( i_{c}-T_{c} \right)T_{bc}^{2})}$ Equation 5

$s.e\left( r_{bc}f_{b}f_{c} \right)\approx\frac{1}{a_{R}}\sqrt{[\frac{{{s.e}_{K_{c}}}^{2}}{{y_{c}}^{2}}\left( \frac{1}{i_{c}}+a_{R}r_{bc}h_{b}h_{c}\left( i_{c}-T_{c} \right) \right)^{2}+\frac{1}{i_{c}^{2}}(\frac{{{s.e}_{K_{b}}}^{2}}{{y_{b}}^{2}}+\frac{{{s.e}_{K_{bc}}}^{2}}{{y_{bc}}^{2}})}]$ Equation 6

Here_,_ $T_{b}$, $T_{bc}$ and $T_{c}$are the thresholds of disease $b$ in the general population, disease $b$ in the relatives of individuals affected by disease $c$, and disease $c$ in the general population, respectively. Furthermore, $i_{c}$ is the mean liability of the individuals affected by disease $c$.

Co-familiality was estimated for both first- and second-degree relatives. To obtain overall co-familiality estimates, we weighted the first- and second-degree relative estimates by the inverse of their sampling variances.

Lastly, we obtain the familial correlation ($r_{f}$) and its approximate s.e. through

$r_{f}=\frac{r_{bc}f_{b}f_{c}}{f_{b}f_{c}}$ Equation 7

$s.e\left( r_{f} \right)\approx r_{f}\sqrt{\frac{{s.e\left( r_{bc}f_{b}f_{c} \right)}^{2}}{{r_{bc}f_{b}f_{c}}^{2}}+\frac{{(f_{b}f_{c}\sqrt{\frac{0.25\times{(f_{b}^{2}f_{c}^{2}\sqrt{\frac{{s.e\left( f_{b}^{2} \right)}^{2}}{{f_{b}^{2}}^{2}}+\frac{{s.e\left( f_{c}^{2} \right)}^{2}}{{f_{c}^{2}}^{2}}})}^{2}}{({f_{b}^{2}f_{c}^{2})}^{2}}})}^{2}}{f_{b}^{2}f_{c}^{2}}}$ Equation 8

**Supplementary references**

American Psychiatric Association (Ed.). (2000). *Diagnostic and Statistical Manual of Mental Disorders Fourth Edition Text Revision (DSM-IV-TR)* (4. ed., 9. print). Washington, DC.

Arel-Bundock, V. (2024). *marginaleffects: Predictions, Comparisons, Slopes, Marginal Means, and Hypothesis Tests*. Retrieved from https://marginaleffects.com/

Falconer, D. S. (1965). The inheritance of liability to certain diseases, estimated from the incidence among relatives. *Annals of Human Genetics*, *29*(1), 51–76. doi: 10.1111/j.1469-1809.1965.tb00500.x

Reich, T., James, J. W., & Morris, C. A. (1972). The use of multiple thresholds in determining the mode of transmission of semi-continuous traits. *Annals of Human Genetics*, *36*(2), 163–184. doi: 10.1111/j.1469-1809.1972.tb00767.x

Risch, N. (1990). Linkage strategies for genetically complex traits. I. Multilocus models. *American Journal of Human Genetics*, *46*(2), 222–228.

Sheehan, D. V., Lecrubier, Y., Sheehan, K. H., Amorim, P., Janavs, J., Weiller, E., … Dunbar, G. C. (1998). The Mini-International Neuropsychiatric Interview (M.I.N.I.): The Development and Validation of a Structured Diagnostic Psychiatric Interview for DSM-IV and ICD-10. *The Journal of Clinical Psychiatry*, *59*(suppl 20), 11980.

van Loo, H. M., Beijers, L., Wieling, M., de Jong, T. R., Schoevers, R. A., & Kendler, K. S. (2023). Prevalence of internalizing disorders, symptoms, and traits across age using advanced nonlinear models. *Psychological Medicine*, *53*(1), 78–87. doi: 10.1017/S0033291721001148

van Loo, H. M., Maeder, L., Bos, M., & Rosmalen, J. G. M. (Manuscript under review). *Prevalence of chronic fatigue syndrome (ME/CFS), fibromyalgia, and irritable bowel syndrome across age and sex using advanced nonlinear models*.

Wray, N. R., & Gottesman, I. I. (2012). Using Summary Data from the Danish National Registers to Estimate Heritabilities for Schizophrenia, Bipolar Disorder, and Major Depressive Disorder. *Frontiers in Genetics*, *3*, 118. doi: 10.3389/fgene.2012.00118
